# Supplementary material for: Chorus wave power at the strong diffusion limit overcomes electron losses due to strong diffusion
Source: Nat Commun. 2024 Feb 27;15:1800. doi: 10.1038/s41467-024-45967-9 (PMC10899562; doi:10.1038/s41467-024-45967-9)
Supplement: Supplementary file 2 — Supplementary Information [file 41467_2024_45967_MOESM2_ESM.docx]

Supplementary Material for Article Titled:

Chorus wave power at the strong diffusion limit overcomes electron losses due to strong diffusion

T. A. Daggitt^1,2^*, R. B. Horne^1^, S. A. Glauert^1^, G Del Zanna^2^, J. M. Albert^3^

^1^British Antarctic Survey, Cambridge, UK

^2^Department of Applied Maths and Theoretical Physics, University of Cambridge, Cambridge, UK

^3^Air Force Research Laboratory, Kirtland AFB, NM, USA

*thoggi18@bas.ac.uk

**Supplementary Fig. 1 Causes of flat pitch angle distributions in POES data**

**
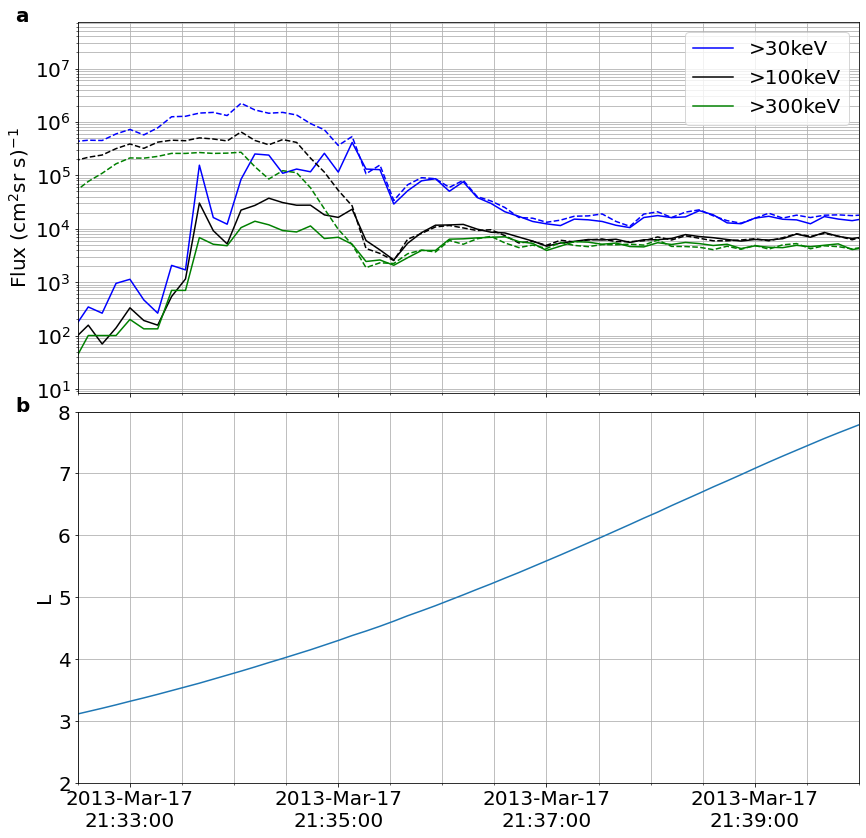
**

**Figure 1: POES N19 data from the 2013 St Patrick’s day storm.** **a** The directional electron flux from the 0^o^ telescope (solid lines) and the 90^o^ (dashed lines) telescope against time. **b** The unitless POES N19 McIlwain L shell against time.

Supplementary figure 1 shows the directional, integrated electron flux for the three energy channels of the POES N19 MEPED instrument for 7 minutes during the 2013 St Patrick’s day storm. At 21:37:30 UTC, all three energy channels simultaneously show near-equal trapped and precipitating flux, as the satellite crosses L ~ 4.5. The additional precipitation here is likely driven by the satellite moving onto a field line where strong chorus waves are present as it cross the plasmapause, rather than field line curvature scattering. In the case of field line curvature scattering the three energy channels would reach equal trapped and precipitating flux at different L values as the radius of curvature of the field line approaches the electron gyroradius at each energy.

**Supplementary Fig. 2 POES observations of an empty loss cone**


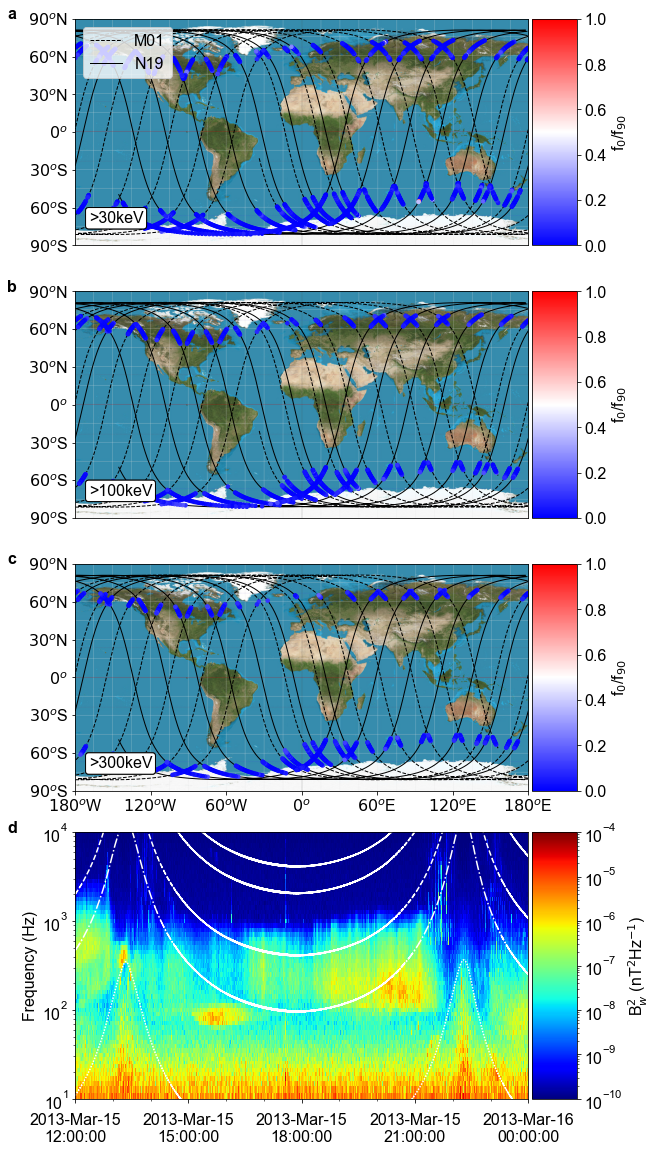


**Figure 2: VAP-A and POES M01 and N19 data prior to the storm. a,b,c** POES data from 12:00:00 March 15^th^ to 00:00:00 March 16^th^ 2013 UTC, showing the ratio between the fluxes from the 0^o^ and 90^o^ telescopes for each POES satellite for each integral energy channel along the track of the satellite (black lines). **d** VAP-A EMFISIS data for the same period, showing no observations of chorus wave power, with the white lines showing 1, 0.5 and 0.1 times the electron gyrofrequency, the lower hybrid resonance frequency and the proton gyrofrequency in order from highest to lowest.

The ratio of trapped to precipitating fluxes recorded by the POES satellites two days prior to the storm is shown in supplementary figure 2, along with simultaneous observations of the wave frequency distribution. This demonstrates a quiet period in which VAP-A does not observe any chorus emissions, and the ratio of trapped to precipitating flux observed by the POES satellites remains below one, showing a steep gradient in flux across the loss cone.


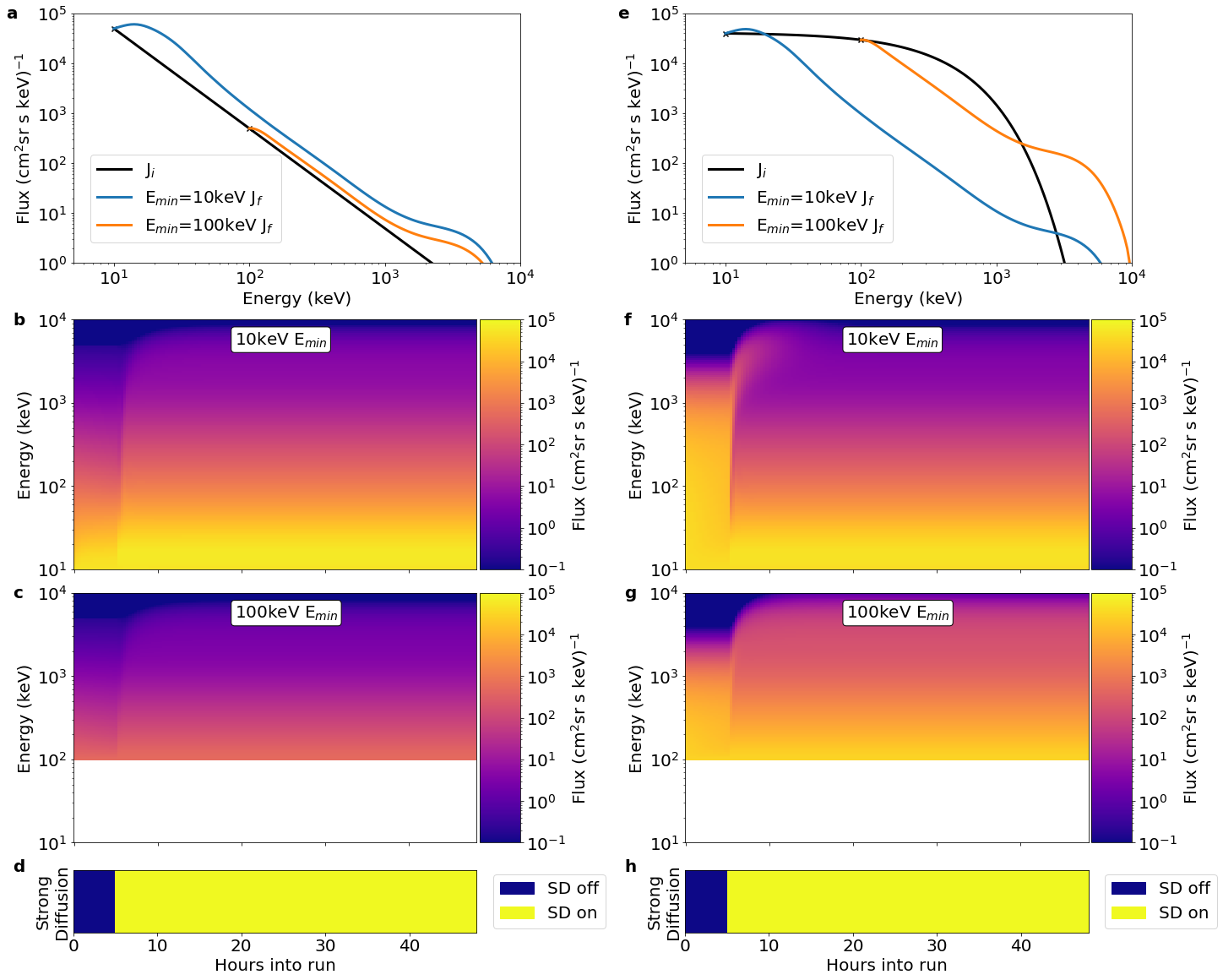
**Supplementary Fig. 3 Effect of moving the low energy boundary**

**Figure 3: BAS-RBM 2D simulations showing the effects of moving the low energy boundary to lower energy.** **a** A comparison of the initial energy spectrum (J_i_) in black, as well as the energy spectrum after 48 hours, (J_f_), for simulations with lower boundaries at 10 and 100keV. **b,c** The flux at different energies during these simulations. **d** Times coloured in blue or yellow show whether the strong diffusion scaling is turned on or off respectively. **a,b,c,d** use a power law initial energy spectrum, **e,f,g,h** show the same data for an exponential initial energy spectrum.

Supplementary figure 3 shows the effect of moving the low energy boundary to a lower energy by moving it along a fixed energy spectrum used as the initial condition. This can either increase or decrease the acceleration of electrons to MeV energies. This is because the steady state energy gradient is fixed by the diffusion coefficients at each energy. A steep gradient in the initial condition between 10 and 100keV will lead to increases in MeV flux, whereas a shallower gradient can lead to a decrease in MeV flux when comparing simulations with low energy boundaries at 10 and 100keV. Moving the low energy boundary in this way leaves the shape of the steady state spectrum largely unchanged and only scales the spectrum up or down in flux.

**
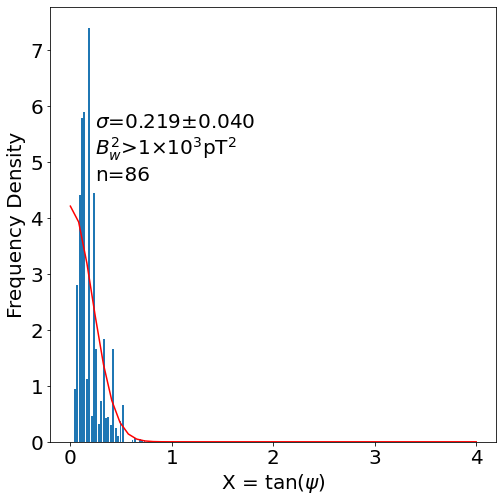
Supplementary Fig. 4 Chorus wave normal angles from EMFISIS data**

**Figure 4: Distribution of lower band chorus wave normal angles during the event.** Wave normal angles, ψ, are recorded by the EMFISIS instrument on board VAP-A. Here observations at wave powers B_w_^2^ > 1×10^3^pT^2^ from 21:00:00 17^th^ – 22:00:00 18^th^ March 2013 UTC, weighted by B_w_^2^ are shown. The red line shows a gaussian fit to the data, with the standard deviation given by σ.

Supplementary figure 4 shows the distribution of wave normal angles for observations with B_w_^2^ > 1×10^3^pT^2^ from the EMFISIS instrument during the event shown in figure 1. The width of this distribution in the tangent of the observed wave normal angle was used to calculate the diffusion coefficients shown in figure 1b. Alterations of the wave normal angle distribution width used in the calculation had negligeable effect on the diffusion coefficients near the loss cone.
